# Sources of antibiotics for acute respiratory infection in children aged <5 years children in South Asia: A multicountry study

**DOI:** 10.1017/ash.2023.254

**Published:** 2023-09-29

**Authors:** Md Abdullah Al Jubayer Biswas, Mohammad Riashad Monjur, South Eastern Sydney Local Health District, Md. Zakiul Hassan, Nusrat Homaira

## Abstract

**Background:** In South Asia, a region of almost 2 billion people across 8 countries, acute respiratory infections (ARIs) are associated with significant morbidity and mortality in children aged <5 years. Although ~80% of ARIs are due to viral etiology and are often self-limiting, they remain the single largest reason for antibiotic use in children aged <5 years in South Asia. We investigated the sources and dispensing pattern of antibiotics for ARIs in children aged <5 years in South Asia. **Methods:** We analyzed nationally representative, population-based, publicly available household survey data from 6 South Asian countries’ Demographic and Health Surveys (DHS): Afghanistan, Bangladesh, India, Maldives, Nepal, and Pakistan. The outcome of interest was the source of antibiotics for children aged <5 years who reportedly had symptoms compatible with ARI (cough, fever, and runny nose) and had received antibiotics for the ARI episode in the 2 weeks preceding the survey. We used a generalized estimating equation with an exchangeable correlation structure to account for country-specific cluster-level correlation to estimate the odds of sources of antibiotics usage. Models were adjusted for age, sex, type of place of residence, wealth index, and parents’ education. To analyze the data, we used the sample weight supplied by the DHS to ensure that our results appropriately reflect the target population in each of the countries studied. **Results:** In total, across the 6 South Asian countries, 24,104 children aged <5 years had symptoms of ARI, 7,587 (31%; 95% CI, 30–33) from received antibiotics. A higher proportion of antibiotic usage for ARIs episodes occurred in Afghanistan (66%), followed by Maldives (53%), Pakistan (45%), and Nepal (43%). Regarding the source of antibiotics, a higher proportion of antibiotics was obtained from the private medical sector in India, followed by unqualified sources in Bangladesh, and the public sector in Afghanistan. Our adjusted multivariable analysis revealed that, in comparison to the public sector, participants were 2.6 times (aOR, 2.6; 95% CI, 1.6–4.3) more likely to receive antibiotics from private medical sector drug sources in Nepal and 1.3 times more likely (aOR, 1.3; 95% CI, 1.1–1.5) in Afghanistan. **Conclusions:** In South Asian countries, the private medical sector was the most common primary source of antibiotics for children with ARIs. Targeted efforts to create awareness around antibiotic dispensing and guidelines to improve practices may curtail the use of antibiotics for ARIs in children aged <5 years in South Asia.

**Disclosures:** None

